# Big data analytics in healthcare: promise and potential

**DOI:** 10.1186/2047-2501-2-3

**Published:** 2014-02-07

**Authors:** Wullianallur Raghupathi, Viju Raghupathi

**Affiliations:** Graduate School of Business, Fordham University, 113 W. 60th Street, 10023 New York, NY USA; Brooklyn College, City University of New York, Brooklyn, NY USA

**Keywords:** Big data, Analytics, Hadoop, Healthcare, Framework, Methodology

## Abstract

**Objective:**

To describe the promise and potential of big data analytics in healthcare.

**Methods:**

The paper describes the nascent field of big data analytics in healthcare, discusses the benefits, outlines an architectural framework and methodology, describes examples reported in the literature, briefly discusses the challenges, and offers conclusions.

**Results:**

The paper provides a broad overview of big data analytics for healthcare researchers and practitioners.

**Conclusions:**

Big data analytics in healthcare is evolving into a promising field for providing insight from very large data sets and improving outcomes while reducing costs. Its potential is great; however there remain challenges to overcome.

## Introduction

The healthcare industry historically has generated large amounts of data, driven by record keeping, compliance & regulatory requirements, and patient care [1]. While most data is stored in hard copy form, the current trend is toward rapid digitization of these large amounts of data. Driven by mandatory requirements and the potential to improve the quality of healthcare delivery meanwhile reducing the costs, these massive quantities of data (known as ‘big data’) hold the promise of supporting a wide range of medical and healthcare functions, including among others clinical decision support, disease surveillance, and population health management [2–5]. Reports say data from the U.S. healthcare system alone reached, in 2011, 150 exabytes. At this rate of growth, big data for U.S. healthcare will soon reach the zettabyte (10^21^ gigabytes) scale and, not long after, the yottabyte (10^24^ gigabytes) [6]. Kaiser Permanente, the California-based health network, which has more than 9 million members, is believed to have between 26.5 and 44 petabytes of potentially rich data from EHRs, including images and annotations [6].

By definition, big data in healthcare refers to electronic health data sets so large and complex that they are difficult (or impossible) to manage with traditional software and/or hardware; nor can they be easily managed with traditional or common data management tools and methods [7]. Big data in healthcare is overwhelming not only because of its volume but also because of the diversity of data types and the speed at which it must be managed [7]. The totality of data related to patient healthcare and well-being make up “big data” in the healthcare industry. It includes clinical data from CPOE and clinical decision support systems (physician’s written notes and prescriptions, medical imaging, laboratory, pharmacy, insurance, and other administrative data); patient data in electronic patient records (EPRs); machine generated/sensor data, such as from monitoring vital signs; social media posts, including Twitter feeds (so-called tweets) [8], blogs [9], status updates on Facebook and other platforms, and web pages; and less patient-specific information, including emergency care data, news feeds, and articles in medical journals.

For the big data scientist, there is, amongst this vast amount and array of data, opportunity. By discovering associations and understanding patterns and trends within the data, big data analytics has the potential to improve care, save lives and lower costs. Thus, big data analytics applications in healthcare take advantage of the explosion in data to extract insights for making better informed decisions [10–12], and as a research category are referred to as, no surprise here, big data analytics in healthcare [13–15]. When big data is synthesized and analyzed—and those aforementioned associations, patterns and trends revealed—healthcare providers and other stakeholders in the healthcare delivery system can develop more thorough and insightful diagnoses and treatments, resulting, one would expect, in higher quality care at lower costs and in better outcomes overall [12]. The potential for big data analytics in healthcare to lead to better outcomes exists across many scenarios, for example: by analyzing patient characteristics and the cost and outcomes of care to identify the most clinically and cost effective treatments and offer analysis and tools, thereby influencing provider behavior; applying advanced analytics to patient profiles (e.g., segmentation and predictive modeling) to proactively identify individuals who would benefit from preventative care or lifestyle changes; broad scale disease profiling to identify predictive events and support prevention initiatives; collecting and publishing data on medical procedures, thus assisting patients in determining the care protocols or regimens that offer the best value; identifying, predicting and minimizing fraud by implementing advanced analytic systems for fraud detection and checking the accuracy and consistency of claims; and, implementing much nearer to real-time, claim authorization; creating new revenue streams by aggregating and synthesizing patient clinical records and claims data sets to provide data and services to third parties, for example, licensing data to assist pharmaceutical companies in identifying patients for inclusion in clinical trials. Many payers are developing and deploying mobile apps that help patients manage their care, locate providers and improve their health. Via analytics, payers are able to monitor adherence to drug and treatment regimens and detect trends that lead to individual and population wellness benefits [12, 16–18].

This article provides an overview of big data analytics in healthcare as it is emerging as a discipline. First, we define and discuss the various advantages and characteristics of big data analytics in healthcare. Then we describe the architectural framework of big data analytics in healthcare. Third, the big data analytics application development methodology is described. Fourth, we provide examples of big data analytics in healthcare reported in the literature. Fifth, the challenges are identified. Lastly, we offer conclusions and future directions.

### Big data analytics in healthcare

Health data volume is expected to grow dramatically in the years ahead [6]. In addition, healthcare reimbursement models are changing; meaningful use and pay for performance are emerging as critical new factors in today’s healthcare environment. Although profit is not and should not be a primary motivator, it is vitally important for healthcare organizations to acquire the available tools, infrastructure, and techniques to leverage big data effectively or else risk losing potentially millions of dollars in revenue and profits [19].

What exactly is big data? A report delivered to the U.S. Congress in August 2012 defines big data as “large volumes of high velocity, complex, and variable data that require advanced techniques and technologies to enable the capture, storage, distribution, management and analysis of the information” [6]. Big data encompasses such characteristics as variety, velocity and, with respect specifically to healthcare, veracity [20–23]. Existing analytical techniques can be applied to the vast amount of existing (but currently unanalyzed) patient-related health and medical data to reach a deeper understanding of outcomes, which then can be applied at the point of care. Ideally, individual and population data would inform each physician and her patient during the decision-making process and help determine the most appropriate treatment option for that particular patient.

### Advantages to healthcare

By digitizing, combining and effectively using big data, healthcare organizations ranging from single-physician offices and multi-provider groups to large hospital networks and accountable care organizations stand to realize significant benefits [2]. Potential benefits include detecting diseases at earlier stages when they can be treated more easily and effectively; managing specific individual and population health and detecting health care fraud more quickly and efficiently. Numerous questions can be addressed with big data analytics. Certain developments or outcomes may be predicted and/or estimated based on vast amounts of historical data, such as length of stay (LOS); patients who will choose elective surgery; patients who likely will not benefit from surgery; complications; patients at risk for medical complications; patients at risk for sepsis, MRSA, C. difficile, or other hospital-acquired illness; illness/disease progression; patients at risk for advancement in disease states; causal factors of illness/disease progression; and possible co-morbid conditions (EMC Consulting). McKinsey estimates that big data analytics can enable more than $300 billion in savings per year in U.S. healthcare, two thirds of that through reductions of approximately 8% in national healthcare expenditures. Clinical operations and R & D are two of the largest areas for potential savings with $165 billion and $108 billion in waste respectively [24]. McKinsey believes big data could help reduce waste and inefficiency in the following three areas:

***Clinical operations***: Comparative effectiveness research to determine more clinically relevant and cost-effective ways to diagnose and treat patients.

***Research & development***: 1) predictive modeling to lower attrition and produce a leaner, faster, more targeted R & D pipeline in drugs and devices; 2) statistical tools and algorithms to improve clinical trial design and patient recruitment to better match treatments to individual patients, thus reducing trial failures and speeding new treatments to market; and 3) analyzing clinical trials and patient records to identify follow-on indications and discover adverse effects before products reach the market.

*Public health*: 1) analyzing disease patterns and tracking disease outbreaks and transmission to improve public health surveillance and speed response; 2) faster development of more accurately targeted vaccines, e.g., choosing the annual influenza strains; and, 3) turning large amounts of data into actionable information that can be used to identify needs, provide services, and predict and prevent crises, especially for the benefit of populations [24].

In addition, [14] suggests big data analytics in healthcare can contribute to

*Evidence-based medicine*: Combine and analyze a variety of structured and unstructured data-EMRs, financial and operational data, clinical data, and genomic data to match treatments with outcomes, predict patients at risk for disease or readmission and provide more efficient care;

*Genomic analytics*: Execute gene sequencing more efficiently and cost effectively and make genomic analysis a part of the regular medical care decision process and the growing patient medical record [25];

*Pre-adjudication fraud analysis*: Rapidly analyze large numbers of claim requests to reduce fraud, waste and abuse;

*Device/remote monitoring*: Capture and analyze in real-time large volumes of fast-moving data from in-hospital and in-home devices, for safety monitoring and adverse event prediction;

*Patient profile analytics*: Apply advanced analytics to patient profiles (e.g., segmentation and predictive modeling) to identify individuals who would benefit from proactive care or lifestyle changes, for example, those patients at risk of developing a specific disease (e.g., diabetes) who would benefit from preventive care [14].

According to [16], areas in which enhanced data and analytics yield the greatest results include: pinpointing patients who are the greatest consumers of health resources or at the greatest risk for adverse outcomes; providing individuals with the information they need to make informed decisions and more effectively manage their own health as well as more easily adopt and track healthier behaviors; identifying treatments, programs and processes that do not deliver demonstrable benefits or cost too much; reducing readmissions by identifying environmental or lifestyle factors that increase risk or trigger adverse events [26] and adjusting treatment plans accordingly; improving outcomes by examining vitals from at-home health monitors; managing population health by detecting vulnerabilities within patient populations during disease outbreaks or disasters; and bringing clinical, financial and operational data together to analyze resource utilization productively and in real time [16].

### The 4 “Vs” of big data analytics in healthcare

Like big data in healthcare, the analytics associated with big data is described by three primary characteristics: volume, velocity and variety (http://www-01.ibm.com/software/data/bigdata/). Over time, health-related data will be created and accumulated continuously, resulting in an incredible *volume* of data. The already daunting volume of existing healthcare data includes personal medical records, radiology images, clinical trial data FDA submissions, human genetics and population data genomic sequences, etc. Newer forms of big data, such as 3D imaging, genomics and biometric sensor readings, are also fueling this exponential growth.

Fortunately, advances in data management, particularly virtualization and cloud computing, are facilitating the development of platforms for more effective capture, storage and manipulation of large volumes of data [4]. Data is accumulated in real-time and at a rapid pace, or *velocity*. The constant flow of new data accumulating at unprecedented rates presents new challenges. Just as the volume and variety of data that is collected and stored has changed, so too has the velocity at which it is generated and that is necessary for retrieving, analyzing, comparing and making decisions based on the output.

Most healthcare data has been traditionally static—paper files, x-ray films, and scripts. Velocity of mounting data increases with data that represents regular monitoring, such as multiple daily diabetic glucose measurements (or more continuous control by insulin pumps), blood pressure readings, and EKGs. Meanwhile, in many medical situations, constant real-time data (trauma monitoring for blood pressure, operating room monitors for anesthesia, bedside heart monitors, etc.) can mean the difference between life and death.

Future applications of real-time data, such as detecting infections as early as possible, identifying them swiftly and applying the right treatments (not just broad-spectrum antibiotics) could reduce patient morbidity and mortality and even prevent hospital outbreaks. Already, real-time streaming data monitors neonates in the ICU, catching life-threatening infections sooner [6]. The ability to perform real-time analytics against such high-volume data in motion and across all specialties would revolutionize healthcare [4]. Therein lies *variety*.

As the nature of health data has evolved, so too have analytics techniques scaled up to the complex and sophisticated analytics necessary to accommodate *volume*, *velocity* and *variety*. Gone are the days of data collected exclusively in electronic health records and other structured formats. Increasingly, the data is in multimedia format and unstructured. The enormous *variety* of data—structured, unstructured and semi-structured—is a dimension that makes healthcare data both interesting and challenging.

Structured data is data that can be easily stored, queried, recalled, analyzed and manipulated by machine. Historically, in healthcare, structured and semi-structured data includes instrument readings and data generated by the ongoing conversion of paper records to electronic health and medical records. Historically, the point of care generated unstructured data: office medical records, handwritten nurse and doctor notes, hospital admission and discharge records, paper prescriptions, radiograph films, MRI, CT and other images.

Already, new data streams—structured and unstructured—are cascading into the healthcare realm from fitness devices, genetics and genomics, social media research and other sources. But relatively little of this data can presently be captured, stored and organized so that it can be manipulated by computers and analyzed for useful information. Healthcare applications in particular need more efficient ways to combine and convert varieties of data including automating conversion from structured to unstructured data.

The structured data in EMRs and EHRs include familiar input record fields such as patient name, data of birth, address, physician’s name, hospital name and address, treatment reimbursement codes, and other information easily coded into and handled by automated databases. The need to field-code data at the point of care for electronic handling is a major barrier to acceptance of EMRs by physicians and nurses, who lose the natural language ease of entry and understanding that handwritten notes provide. On the other hand, most providers agree that an easy way to reduce prescription errors is to use digital entries rather than handwritten scripts.

The potential of big data in healthcare lies in combining traditional data with new forms of data, both individually and on a population level. We are already seeing data sets from a multitude of sources support faster and more reliable research and discovery. If, for example, pharmaceutical developers could integrate population clinical data sets with genomics data, this development could facilitate those developers gaining approvals on more and better drug therapies more quickly than in the past *and*, more importantly, expedite distribution to the right patients [4]. The prospects for all areas of healthcare are infinite.

Some practitioners and researchers have introduced a fourth characteristic, *veracity,* or ‘data assurance’. That is, the big data, analytics and outcomes are error-free and credible. Of course, veracity is the goal, not (yet) the reality. Data quality issues are of acute concern in healthcare for two reasons: life or death decisions depend on having the accurate information, and the quality of healthcare data, especially unstructured data, is highly variable and all too often incorrect. (Inaccurate “translations” of poor handwriting on prescriptions are perhaps the most infamous example).

Veracity assumes the simultaneous scaling up in granularity and performance of the architectures and platforms, algorithms, methodologies and tools to match the demands of big data. The analytics architectures and tools for structured and unstructured big data are very different from traditional business intelligence (BI) tools. They are necessarily of industrial strength. For example, big data analytics in healthcare would be executed in distributed processing across several servers (“nodes”), utilizing the paradigm of parallel computing and ‘divide and process’ approach. Likewise, models and techniques—such as data mining and statistical approaches, algorithms, visualization techniques—need to take into account the characteristics of big data analytics. Traditional data management assumes that the warehoused data is certain, clean, and precise.

Veracity in healthcare data faces many of the same issues as in financial data, especially on the payer side: Is this the correct patient/hospital/payer/reimbursement code/dollar amount? Other veracity issues are unique to healthcare: Are diagnoses/treatments/prescriptions/procedures/outcomes captured correctly?

Improving coordination of care, avoiding errors and reducing costs depend on high-quality data, as do advances in drug safety and efficacy, diagnostic accuracy and more precise targeting of disease processes by treatments. But increased variety and high velocity hinder the ability to cleanse data before analyzing it and making decisions, magnifying the issue of data “trust” [4].

The ‘4Vs’ are an appropriate starting point for a discussion about big data analytics in healthcare. But there are other issues to consider, such as the number of architectures and platforms, and the dominance of the open source paradigm in the availability of tools. Consider, too, the challenge of developing methodologies and the need for user-friendly interfaces. While the overall cost of hardware and software is declining, these issues have to be addressed to harness and maximize the potential of big data analytics in healthcare.

### Architectural framework

The conceptual framework for a big data analytics project in healthcare is similar to that of a traditional health informatics or analytics project. The key difference lies in how processing is executed. In a regular health analytics project, the analysis can be performed with a business intelligence tool installed on a stand-alone system, such as a desktop or laptop. Because big data is by definition large, processing is broken down and executed across multiple nodes. The concept of distributed processing has existed for decades. What is relatively new is its use in analyzing very large data sets as healthcare providers start to tap into their large data repositories to gain insight for making better-informed health-related decisions. Furthermore, open source platforms such as Hadoop/MapReduce, available on the cloud, have encouraged the application of big data analytics in healthcare.

**Figure 1:**
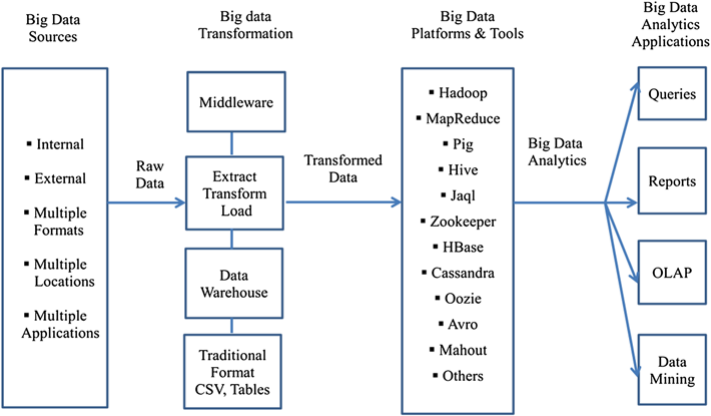
**An applied conceptual architecture of big data analytics.**

While the algorithms and models are similar, the user interfaces of traditional analytics tools and those used for big data are entirely different; traditional health analytics tools have become very user friendly and transparent. Big data analytics tools, on the other hand, are extremely complex, programming intensive, and require the application of a variety of skills. They have emerged in an ad hoc fashion mostly as open-source development tools and platforms, and therefore they lack the support and user-friendliness that vendor-driven proprietary tools possess. As Figure 1 indicates, the complexity begins with the data itself.

Big data in healthcare can come from internal (e.g., electronic health records, clinical decision support systems, CPOE, etc.) and external sources (government sources, laboratories, pharmacies, insurance companies & HMOs, etc.), often in multiple formats (flat files, .csv, relational tables, ASCII/text, etc.) and residing at multiple locations (geographic as well as in different healthcare providers’ sites) in numerous legacy and other applications (transaction processing applications, databases, etc.). Sources and data types include:

1. Web and social media data: Clickstream and interaction data from Facebook, Twitter, LinkedIn, blogs, and the like. It can also include health plan websites, smartphone apps, etc. [6].
2. Machine to machine data: readings from remote sensors, meters, and other vital sign devices [6].
3. Big transaction data: health care claims and other billing records increasingly available in semi-structured and unstructured formats [6].
4. Biometric data: finger prints, genetics, handwriting, retinal scans, x-ray and other medical images, blood pressure, pulse and pulse-oximetry readings, and other similar types of data [6].
5. Human-generated data: unstructured and semi-structured data such as EMRs, physicians notes, email, and paper documents [6].

For the purpose of big data analytics, this data has to be pooled. In the second component the data is in a ‘raw’ state and needs to be processed or transformed, at which point several options are available. A service-oriented architectural approach combined with web services (middleware) is one possibility [27]. The data stays raw and services are used to call, retrieve and process the data. Another approach is data warehousing wherein data from various sources is aggregated and made ready for processing, although the data is not available in real-time. Via the steps of extract, transform, and load (ETL), data from diverse sources is cleansed and readied. Depending on whether the data is structured or unstructured, several data formats can be input to the big data analytics platform.

In this next component in the conceptual framework, several decisions are made regarding the data input approach, distributed design, tool selection and analytics models. Finally, on the far right, the four typical applications of big data analytics in healthcare are shown. These include queries, reports, OLAP, and data mining. Visualization is an overarching theme across the four applications. Drawing from such fields as statistics, computer science, applied mathematics and economics, a wide variety of techniques and technologies has been developed and adapted to aggregate, manipulate, analyze, and visualize big data in healthcare.

The most significant platform for big data analytics is the open-source distributed data processing platform Hadoop (Apache platform), initially developed for such routine functions as aggregating web search indexes. It belongs to the class “NoSQL” technologies—others include CouchDB and MongoDB—that evolved to aggregate data in unique ways. Hadoop has the potential to process extremely large amounts of data mainly by allocating partitioned data sets to numerous servers (nodes), each of which solves different parts of the larger problem and then integrates them for the final result [28–31]. Hadoop can serve the twin roles of data organizer and analytics tool. It offers a great deal of potential in enabling enterprises to harness the data that has been, until now, difficult to manage and analyze. Specifically, Hadoop makes it possible to process extremely large volumes of data with various structures or no structure at all. But Hadoop can be challenging to install, configure and administer, and individuals with Hadoop skills are not easily found. Furthermore, for these reasons, it appears organizations are not quite ready to embrace Hadoop completely. The surrounding ecosystem of additional platforms and tools supports the Hadoop distributed platform [30, 31]. These are summarized in Table 1.

**Table 1:** **Platforms & tools for big data analytics in healthcare**

Platform/Tool	Description
The Hadoop Distributed File System (HDFS)	HDFS enables the underlying storage for the Hadoop cluster. It divides the data into smaller parts and distributes it across the various servers/nodes.
MapReduce	MapReduce provides the interface for the distribution of sub-tasks and the gathering of outputs. When tasks are executed, MapReduce tracks the processing of each server/node.
PIG and PIG Latin (Pig and PigLatin)	Pig programming language is configured to assimilate all types of data (structured/unstructured, etc.). It is comprised of two key modules: the language itself, called PigLatin, and the runtime version in which the PigLatin code is executed.
Hive	Hive is a runtime Hadoop support architecture that leverages Structure Query Language (SQL) with the Hadoop platform. It permits SQL programmers to develop Hive Query Language (HQL) statements akin to typical SQL statements.
Jaql	Jaql is a functional, declarative query language designed to process large data sets. To facilitate parallel processing, Jaql converts “‘high-level’ queries into ‘low-level’ queries” consisting of MapReduce tasks.
Zookeeper	Zookeeper allows a centralized infrastructure with various services, providing synchronization across a cluster of servers. Big data analytics applications utilize these services to coordinate parallel processing across big clusters.
HBase	HBase is a column-oriented database management system that sits on top of HDFS. It uses a non-SQL approach.
Cassandra	Cassandra is also a distributed database system. It is designated as a top-level project modeled to handle big data distributed across many utility servers. It also provides reliable service with no particular point of failure (http://en.wikipedia.org/wiki/Apache_Cassandra) and it is a NoSQL system.
Oozie	Oozie, an open source project, streamlines the workflow and coordination among the tasks.
Lucene	The Lucene project is used widely for text analytics/searches and has been incorporated into several open source projects. Its scope includes full text indexing and library search for use within a Java application.
Avro	Avro facilitates data serialization services. Versioning and version control are additional useful features.
Mahout	Mahout is yet another Apache project whose goal is to generate free applications of distributed and scalable machine learning algorithms that support big data analytics on the Hadoop platform.

Numerous vendors—including AWS, Cloudera, Hortonworks, and MapR Technologies—distribute open-source Hadoop platforms [29]. Many proprietary options are also available, such as IBM’s BigInsights. Further, many of these platforms are cloud versions, making them widely available. Cassandra, HBase, and MongoDB, described above, are used widely for the database component. While the available frameworks and tools are mostly open source and wrapped around Hadoop and related platforms, there are numerous trade-offs that developers and users of big data analytics in healthcare must consider. While the development costs may be lower since these tools are open source and free of charge, the downsides are the lack of technical support and minimal security. In the healthcare industry, these are, of course, significant drawbacks, and therefore the trade-offs must be addressed. Additionally, these platforms/tools require a great deal of programming, skills the typical end-user in healthcare may not possess. Furthermore, considering the only recent emergence of big data analytics in healthcare, governance issues including ownership, privacy, security, and standards have yet to be addressed. In the next section we offer an applied big data analytics in healthcare methodology to develop and implement a big data project for healthcare providers.

### Methodology

While several different methodologies are being developed in this rapidly emerging discipline, here we outline one that is practical and hands-on. Table 2 shows the main stages of the methodology. In *Step 1*, the interdisciplinary big data analytics in healthcare team develops a ‘concept statement’. This is a first cut at establishing the need for such a project. The concept statement is followed by a description of the project’s significance. The healthcare organization will note that there are trade-offs in terms of alternative options, cost, scalability, etc. Once the concept statement is approved, the team can proceed to *Step 2*, the proposal development stage. Here, more details are filled in. Based on the concept statement, several questions are addressed: What problem is being addressed? Why is it important and interesting to the healthcare provider? What is the case for a ‘big data’ analytics approach? (Because the complexity and cost of big data analytics are significantly higher compared to traditional analytics approaches, it is important to justify their use). The project team also should provide background information on the problem domain as well as prior projects and research done in this domain.

**Table 2:** **Outline of big data analytics in healthcare methodology**

Step 1	Concept statement
	• Establish need for big data analytics project in healthcare based on the “4Vs”.
Step 2	Proposal
	• What is the problem being addressed?
	• Why is it important and interesting?
	• Why big data analytics approach?
	• Background material
Step 3	Methodology
	• Propositions
	• Variable selection
	• Data collection
	• ETL and data transformation
	• Platform/tool selection
	• Conceptual model
	• Analytic techniques
	-Association, clustering, classification, etc.
	• Results & insight
Step 4	Deployment
	• Evaluation & validation
	• Testing

Source: Adapted from [Raghupathi & Raghupathi, [9]].

Next, in *Step 3*, the steps in the methodology are fleshed out and implemented. The concept statement is broken down into a series of propositions. (Note these are not rigorous as they would be in the case of statistical approaches. Rather, they are developed to help guide the big data analytics process). Simultaneously, the independent and dependent variables or indicators are identified. The data sources, as outlined in Figure 1, are also identified; the data is collected, described, and transformed in preparation for for analytics. A very important step at this point is platform/tool evaluation and selection. There are several options available, as indicated previously, including AWS Hadoop, Cloudera, and IBM BigInsights. The next step is to apply the various big data analytics techniques to the data. This process differs from routine analytics only in that the techniques are scaled up to large data sets. Through a series of iterations and what-if analyses, insight is gained from the big data analytics. From the insight, informed decisions can be made. In *Step 4*, the models and their findings are tested and validated and presented to stakeholders for action. Implementation is a staged approach with feedback loops built in at each stage to minimize risk of failure.

The next section describes several reported big data analytics applications in healthcare. We draw on publicly available material from numerous sources, including vendor sites. In this emerging discipline, there is little independent research to cite. These examples are from secondary sources. Nevertheless, they are illustrative of the potential of big data analytics in healthcare.

### Examples

Premier, the U.S. healthcare alliance network, has more than 2,700 members, hospitals and health systems, 90,000 non-acute facilities and 400,000 physicians and is reported to have data on approximately one in four patients discharged from hospitals. Naturally, the network has assembled a large database of clinical, financial, patient, and supply chain data, with which the network has generated comprehensive and comparable clinical outcome measures, resource utilization reports and transaction level cost data. These outputs have informed decision-making and improved the healthcare processes at approximately 330 hospitals, saving an estimated 29,000 lives and reducing healthcare spending by nearly $7 billion [16]. North York General Hospital, a 450-bed community teaching hospital in Toronto, Canada, reports using real-time analytics to improve patient outcomes and gain greater insight into the operations of healthcare delivery. North York is reported to have implemented a scalable real-time analytics application to provide multiple perspectives, including clinical, administrative, and financial [16]. Another example, reported by IBM, is that of the large, unnamed healthcare provider that is analyzing data in the electronic medical record (EMR) system with the goal of reducing costs and improving patient care. (Data in the EMR include the unstructured data from physician notes, pathology reports and other sources). Big data analytics is used to develop care protocols and case pathways and to assist caregivers in performing customized queries [16]. Another example of big data analytics in healthcare is Columbia University Medical Center’s analysis of “complex correlations” of streams of physiological data related to patients with brain injuries. The goal is to provide medical professionals with critical and timely information to aggressively treat complications. The advanced analytics is reported to diagnose serious complications as much as 48 hours sooner than previously in patients who have suffered a bleeding stroke from a ruptured brain aneurysm [16]. The Rizzoli Orthopedic Institute in Bologna, Italy, is reportedly using advanced analytics to gain a more “granular understanding” of the clinical variations within families whereby individual patients display extreme differences in the severity of their symptoms. This insight is reported to have reduced annual hospitalizations by 30% and the number of imaging tests by 60%. In the long-term, the Institute expects to gain insight into the role of genetic factors to develop treatments [16]. The Hospital for Sick Children (Sick Kids) in Toronto is using analytics to improve the outcomes for infants prone to life-threatening “nosocomial infections”. It is reported that Sick Kids applies advanced analytics to vital-sign data gathered from bedside monitoring devices to identify potential signs infection as early as 24 hours prior to previous methods [6, 16]. Additional examples are reported below.

A recent *New Yorker* magazine article by Atul Gawande, MD described how orthopedic surgeons at Brigham and Women’s Hospital in Boston relied on personal experience along with insight extracted from research on data based on a host of factors critical to the success of joint-replacement surgery to systematically standardize knee joint-replacement surgery. The result: improved outcomes at lower costs. The University of Michigan Health System standardized the administration of blood transfusions using analytics in a similar fashion, combining experience with big data analytics research. This resulted in a 31% reduction in transfusions and $200,000 reduction in expenses per month (reported in [6]). Another example is The National Institute for Health and Clinical Excellence (NICE) of the U.K.’s National Health Service. NICE is reportedly a leader in the analytics of large clinical datasets for exploring the effectiveness of clinical and cost factors in the use of new drugs and/or clinical treatments. The Italian Medicines Agency is also reported to collect and analyze clinical data on the use of expensive new drugs as one goal in a country-level cost-effectiveness program [6]. Another leading example of big data analytics in healthcare is the Department of Veterans Affairs’ (VA) use of applications on its very large data set in an effort to comply with “performance-based accountability framework and disease management practice” [6]. In one very famous example, California-based Kaiser Permanente associated clinical data with cost data to generate a key data set, the analytics of which led to the discovery of adverse drug effects and subsequent withdrawal of Vioxx from the market [6]. Researchers at the Johns Hopkins School of Medicine discovered they could use data from Google Flu Trends to predict sudden increases in flu-related emergency room visits at least a week before warnings from the CDC. Likewise, the analysis of Twitter updates was as accurate as (and two weeks ahead of) official reports at tracking the spread of cholera in Haiti after the January 2010 earthquake [6]. Also reported is an application developed by IBM that predicts the likely outcomes of diabetes patients using patients’ panel data linked to physicians, management protocols, and the overall relationship to population health management averages [6]. In another diabetes application, physicians at Harvard Medical School and Harvard Pilgrim Health Care recently demonstrated the potential of analytics applications to EHR data to identify and group patients with diabetes for public health surveillance. Four years worth of data based on numerous indicators from multiple sources was utilized. The analytics application also differentiated between Type 1 and Type II diabetes [6, 26]. Finally, at Blue Cross Blue Shield of Massachusetts (BCBSMA) there was a “need to embed analytics into business processes to help decision-makers across the business gain insight into financial and medical data and become more proactive”. Several benefits were reported. First, the analytics enabled medical directors to identify high-risk disease groups and act to minimize risk and improve patient outcomes. For example, new preventive treatment protocols could be introduced among patient groups with high cholesterol, thereby fending off heart problems. Also, complex health informatics reports were generated 300% faster than previously, helping BCBSMA service clients more effectively [6].

The next section briefly identifies some of the key challenges in big data analytics in healthcare.

### Challenges

At minimum, a big data analytics platform in healthcare must support the key functions necessary for processing the data. The criteria for platform evaluation may include availability, continuity, ease of use, scalability, ability to manipulate at different levels of granularity, privacy and security enablement, and quality assurance [6, 29, 32]. In addition, while most platforms currently available are open source, the typical advantages and limitations of open source platforms apply. To succeed, big data analytics in healthcare needs to be packaged so it is menu-driven, user-friendly and transparent. Real-time big data analytics is a key requirement in healthcare. The lag between data collection and processing has to be addressed. The dynamic availability of numerous analytics algorithms, models and methods in a pull-down type of menu is also necessary for large-scale adoption. The important managerial issues of ownership, governance and standards have to be considered. And woven through these issues are those of continuous data acquisition and data cleansing. Health care data is rarely standardized, often fragmented, or generated in legacy IT systems with incompatible formats [6]. This great challenge needs to be addressed as well.

## Conclusions

Big data analytics has the potential to transform the way healthcare providers use sophisticated technologies to gain insight from their clinical and other data repositories and make informed decisions. In the future we’ll see the rapid, widespread implementation and use of big data analytics across the healthcare organization and the healthcare industry. To that end, the several challenges highlighted above, must be addressed. As big data analytics becomes more mainstream, issues such as guaranteeing privacy, safeguarding security, establishing standards and governance, and continually improving the tools and technologies will garner attention. Big data analytics and applications in healthcare are at a nascent stage of development, but rapid advances in platforms and tools can accelerate their maturing process.
